# Nutrigenetics and Nutrigenomics Insights into Diabetes Etiopathogenesis

**DOI:** 10.3390/nu6115338

**Published:** 2014-11-21

**Authors:** Genoveva Berná, María Jesús Oliveras-López, Enrique Jurado-Ruíz, Juan Tejedo, Francisco Bedoya, Bernat Soria, Franz Martín

**Affiliations:** 1 Department of Stem Cells, Andalusian Center of Molecular Biology and Regenerative Medicine, University Pablo Olavide (CABIMER-UPO), Seville 41091, Spain; E-Mails: gberamo@upo.es (G.B.); mjolilop@upo.es (M.J.O.-L.); ejurrui@upo.es (E.J.-R.); bernat.soria@cabimer.es (B.S.); 2 Centro de Investigación Biomédica en Red de Diabetes y Enfermedades Metabólicas Asociadas (CIBERDEM), CIBER of Diabetes and Associated Metabolic Diseases, Instituto de Salud Carlos III, Madrid 28029, Spain; E-Mails: juan.tejedo@cabimer.es (J.T.); fbedber@upo.es (F.B.); 3 Department of Cell Therapy and Regenerative Medicine, Andalusian Center of Molecular Biology and Regenerative Medicine, University Pablo Olavide (CABIMER-UPO), Seville 41091, Spain

**Keywords:** diabetes, insulin resistance, pancreatic β cell, gene-nutrient interaction, nutrigenetics, nutrigenomics, epigenetics, miRNAs, nutrients, dietary patterns

## Abstract

Diabetes mellitus (DM) is considered a global pandemic, and the incidence of DM continues to grow worldwide. Nutrients and dietary patterns are central issues in the prevention, development and treatment of this disease. The pathogenesis of DM is not completely understood, but nutrient-gene interactions at different levels, genetic predisposition and dietary factors appear to be involved. Nutritional genomics studies generally focus on dietary patterns according to genetic variations, the role of gene-nutrient interactions, gene-diet-phenotype interactions and epigenetic modifications caused by nutrients; these studies will facilitate an understanding of the early molecular events that occur in DM and will contribute to the identification of better biomarkers and diagnostics tools. In particular, this approach will help to develop tailored diets that maximize the use of nutrients and other functional ingredients present in food, which will aid in the prevention and delay of DM and its complications. This review discusses the current state of nutrigenetics, nutrigenomics and epigenomics research on DM. Here, we provide an overview of the role of gene variants and nutrient interactions, the importance of nutrients and dietary patterns on gene expression, how epigenetic changes and micro RNAs (miRNAs) can alter cellular signaling in response to nutrients and the dietary interventions that may help to prevent the onset of DM.

## 1. Introduction

Diabetes mellitus (DM) is a group of metabolic diseases characterized by hyperglycemia, which results from defects in insulin secretion, insulin activity or both. DM is associated with the dysfunction and failure of different organs, such as the blood vessels, heart and kidneys [1], and this disease is considered a global burden [2]. The International Diabetes Federation’s most recent estimates indicate that 8.3% of adults (382 million individuals) have diabetes, and the number of individuals with this disease is expected to rise beyond 592 million in less than 25 years [2]. The vast majority of cases of DM fall into two broad etiopathogenetic categories: type 1 and type 2 DM (T1DM and T2DM, respectively). T1DM, previously named insulin-dependent diabetes or juvenile-onset diabetes, results from cellular-mediated autoimmune destruction of pancreatic β cells; therefore, patients are dependent on exogenous insulin. Individuals with T1DM are considered to have a genetic predisposition, although environmental factors, such as dietary components, also contribute to T1DM development [3]. Thus, T1DM is the result of a complex interrelation among β cells, the immune system and environmental factors in genetically susceptible individuals [3]. T1DM appears predominately in children and young adults and affects 5%–10% of diabetic patients [2]. T2DM is chronic disorder caused by insulin secretion deficiency and insulin resistance. T2DM is a complex trait that results from the contribution of many genes [4], many environmental factors, including diet [5], and the interactions among these genes and environmental factors. T2DM is more common among individuals aged 40 to 60 years and accounts for most cases of DM (more than 90%) [2].

The incidence rates of both types of DM are increasing and their exact causes are not completely understood. It seems that interactions between multiple genes and environmental factors may play a role. One of these factors is dietary factors. There is evidence supporting the role of nutrient-gene interactions in DM pathophysiology [5]. Thus, a greater understanding of potential gene-nutrient interactions may be relevant for DM prevention and treatment.

Nutrigenetics and nutrigenomics are defined as the science of the effects of genetic variation on dietary responses and the role of nutrients and bioactive food compounds in gene expression, respectively [6]. It is important to note that both terms are closely related but not interchangeable. Nutrigenetics research involves genetic inheritance and its variations in the response to nutrients and dietary patterns [7], whereas nutrigenomics investigations focus on dietary effects on genome stability, epigenome alterations, RNA and miRNA alterations, protein expression and metabolite changes. Both fields depend on advances in genomics, transcriptomics, proteomics and metabolomics, as these high-throughput technologies enable the analysis of many different genes and their variants, metabolites and a large number of nutrients and bioactives present in food and how they affect human metabolism, nutritional homeostasis and molecular events involved in nutrition-related diseases, such as diabetes. However, while the application of these technologies is becoming more accessible, analysis of the complex large data sets that are generated presents multiple challenges.

The aim of the present review was to provide insights regarding the role of nutrient-gene interactions in DM pathogenesis, prevention and treatment. In addition, we explored how an individual’s genetic makeup can affect nutrient metabolism and the response to nutrient intake, potentially leading to DM.

It is important to promote greater research in this field because these findings will provide a framework for the development of genotype-dependent food health promotion strategies and the design of dietetic approaches for the prevention and management of DM. This knowledge has begun to provide evidence where specific targeted nutritional advice, such as following a Mediterranean Diet, helps to decrease cardiovascular risk factors and stroke incidence in people with polymorphisms strongly associated with T2DM [8].

## 2. Nutrigenetics Approximation to DM

Chronic non-communicable diseases (NCD), such as coronary heart disease, high blood pressure, cancer or DM, which account for approximately 60% of global mortality [9], tend to aggregate in families, and the risk among relatives is higher compared to the general population [10]. Families share both genes and environment; however, various families, even those composed of different ethnic groups, may live together in the same cities within a homogenous environment. In these situations, individual genetic variants or inheritances contribute to NCD susceptibility, such as DM, through the modulation of the response to nutrients or diets. In this regard, approximately one decade ago, genome-wide association studies (GWAS) revealed certain genomic variants that predisposed individuals to DM [11]. Furthermore, GWAS results have also highlighted the importance of dietary variables [12].

Genetic variation across the human genome has been recognized as increasingly complex. Single-nucleotide polymorphisms (SNPs) are the most common type of genetic variations dispersed within or outside a gene region in the human genome. Approximately one decade ago, there were more than 10 million SNPs reported in public databases [13]. Genetic polymorphisms are normally identified in at least 1% of the population, and approximately 54% of these variants are not deleterious mutations [14]. In the case of DM, these variants, in general, do not directly cause the disease but alter the risk of developing DM [15].

Recent GWAS have successfully identified more than 40 independent T1DM-associated tagging SNPs; however, the sum of these loci does not fully explain the heritability estimated from familial studies [16]. For example, twin studies have shown that for di-zygotic twins, the pairwise T1DM concordance rate is 10%, whereas for mono-zygotic twins, the concordance rate is approximately 50% [17]. Thus, dietary and other environmental factors also influence T1DM incidence and development. These factors primarily include the use of breast milk *vs.* infant formula [18], highly hydrolyzed infant formula *vs.* conventional infant formula [19], early/late exposure to gluten [20] and vitamin D [21]. Interestingly, a newly diagnosed child fed a gluten-free diet was shown to remain healthy without insulin therapy for 20 months [22].

Over the last five years, several studies have linked diet/nutrients (mainly dietary fiber), gut microbiota and the expression of genes involved in immune responses. It is well known that the diet has a profound effect on the gut microbiota. In mice and humans, microbes respond differently to dietary components, and long-term dietary habits have been linked to the abundance of certain microbial genera [23]. The gut lumen contains large amounts of nutrients that strongly influence the composition of the microbiota, which affects gut immunity. These alterations in gut immunity can precipitate T1DM in individuals prone to T1DM. It has also been observed that diabetes-prone BioBreeding (BBdp) rats housed in specific germ-free (GF) conditions and weaned onto cereal diets displayed an upregulation of the interferon gamma (Ifng) and interleukin 15 (Il15) genes and a downregulation of the forkhead box P3 (Foxp3) gene [24]. Both Ifng and IL-15 are proinflammatory cytokines that promote T1DM in non-obese diabetic (NOD) mice [25], whereas Foxp3 is a master transcription factor that directs the differentiation and function of regulatory T cells and plays a central role in the inhibition of autoimmunity and suppression of physiological immune responses [26]. When BBdp rats were weaned onto cereal diets and housed in specific pathogen-free conditions (allowing gut microbiota growth), the rats also showed an upregulation of the lymphocyte-specific protein tyrosine kinase (Lck) gene [23]. Lck encodes tyrosine kinase/p56, a lymphocyte-specific protein involved in the initiation of T cell activation [27]. Finally, in this last condition, BBdp rats showed decreased expression of the cathelicidin antimicrobial peptide (Camp) gene. CAMP is a multifunctional antimicrobial effector and immunomodulatory host defense factor [28], which may alter the gut microbiota.

Thus, for T1DM, nutrients can modify, alone or through changes in the gut microbiota, the expression of genes involved in the immune response. As a result, these changes may promote autoimmune responses in individuals predisposed to this condition.

Recent advancements in human genetics have led to the identification of a relatively large number of T2DM-associated loci, more than 65 loci, many of which are novel [29] and increase the risk of T2DM by 10%–30%. However, their contribution to disease risk appears to be poor, and their predictive value is small because lifestyle plays a crucial role in T2DM development [30]. Studies that have investigated the gene-lifestyle interactions in T2DM have suggested that the biological effects of genetic predisposition may be partially or nearly completely abolished by a healthy lifestyle or lifestyle modifications [31]. Moreover, the contribution of the many genes and their relationship with numerous environmental factors confounds the common experimental designs used to identify gene-nutrient interactions. Thus, the experimental methods successfully applied to describe the genetic basis of monogenic diseases cannot be applied to complex traits, such as T2DM. To bypass this problem, a method called quantitative trait locus (QTL) analysis has been developed. This methodology allows the identification of regions of chromosomes that contribute to a complex trait [32]. QTLs are identified through statistical analysis of how frequently a region of a chromosome is associated with a measurable phenotype, e.g., plasma insulin levels or the homeostasis model assessment (HOMA) index. Finally, each of the genes within the QTLs may contribute different amounts to the trait. In this regard, SNPs may therefore be associated with small or large contributions to the complex trait [33]; the contribution will vary depending upon gene-nutrient interactions for the gene responsible for the QTL and whether that gene interacts with other genes in the genome.

To date, more than 70 genes have been identified as involved in T2DM, primarily by association analysis [34]. In addition, via GWAS arrays, more than 100 SNPs have been identified for T2DM [35]. From the 50 novel loci associated with T2DM previously identified, more than 40 loci have been associated with T2DM-related traits, including fasting proinsulin, insulin and glucose (Table 1) [36,37,38,39]. However, for T2DM-related traits, such as the HOMA index or pancreatic β cell function, there are virtually no published data examining the relationship between these traits or the genotype and environment interactions. Clinical investigations of some loci have suggested that the genetic components of T2DM risk act preferentially through β cell function [40]. Among all 40 loci associated with T2DM-related traits, only transcription factor-7-like 2 (TCF7L2) was shown to clearly contribute to T2DM risk [41]. Several studies in white European [42], Indian [43], Japanese [44], Mexican American [45] and West African [46] individuals have shown a strong association between TCF7L2 and T2DM. It is also noteworthy that these populations represent the major racial groups with a high prevalence of T2DM. In all populations, TCF7L2 showed a strong association, with the odds of developing T2DM increased by 30%–50% for each allele inherited. This finding indicates an approximately double odds ratio compared to most other diabetes susceptibility polymorphisms. TCF7L2 is a transcription factor involved in the Wnt signaling pathway that is ubiquitously expressed, and it has been observed that TCF7L2 risk alleles result in the overexpression of TCF7L2 in pancreatic β cells. This overexpression causes reduced nutrient-induced insulin secretion, which results in a direct predisposition to T2DM as well as an indirect predisposition via an increase in hepatic glucose production [47].

**Table 1:** Loci for T2DM-related traits identified by GWAS (in order of gene region).

Loci					
NOTCH2	PSMD6	VGEFA	CHCHD9	DCD	CMIP
ADAM30	CACNA1D	CDKAL1	GAS1	HMGA2	WWOX
SLC44A3	PPARG	C6orf57	CAMK1D	TMEM19	SGSM2
SNX7	SYN2	TP53INP1	CDC123	LGR5	SRR
PROX1	ZPLD1	GCK	VPS26A	TSPAN8	HNF1B
CR2	PLS1	CPVL	KIF11	IGF1	LPIN2
PCNXL2	SLC2A2	JAZF1	HHEK	HNF1A	PAPL
BCL11A	PEX5L	DGKB	ADRA2A	TRIAP1	PEPD
THADA	IGF2BP2	ACHE	TCF7L2	SPRY2	GIPR
GCKR	ST6GAL1	GCC1	TCERG1L	C14orf70	HNF4A
ITGB2	PPP2R2C	PAX4	CRY2	ATP10A	HUNK
RBM43	WFS1	KLF14	MADD	C2CD4A	PCBP3
RND3	MAEA	ZMAT4	KCNJ11	C2CD4B	SEZ6L
ITGB6	ZBED3	KCNU1	GALNTL4	VPS13C	DUSP9
RBMS1	AP3B1	CSMD1	LOC72903	LARP6	
GRB14	CETN3	SLC30A8	KCNQ1	HMG20A	
G6PC2	LOC72901	CDKN2A	ARAP1	ZFAND6	
TMEFF2	PCSK1	CDKN2B	MTNR1B	AP3S2	
IRS1	KCNK16	PTRD	BARX2	PRC1	
ADAMTS9	ZFAND3	GLIS3	TMEM45B	FTO	

## 3. Gene-Nutrient or Dietary Pattern Interactions in The Development of T2DM

Recently, several studies have demonstrated the significant effects of genotype by environment interactions on T2DM [48,49]. However, further clarification of the role of these interactions at the genome-wide level could help predict disease risk more accurately and facilitate the development of dietary recommendations to improve prevention and treatment. Moreover, it would be very interesting to identify the specific dietary factors that are the most influential in the variation of a given T2DM-related phenotype and to what extent these dietary factors contribute to the phenotypic variation (Table 2). In particular, the dietary factors considered are macro- and micronutrients, foods and type of diets. A recent review present evidence on the dietary environment and genetics as risk factors for T2DM [50].

**Table 2:** Gene-nutrient or -dietary pattern interactions in the development of T2DM.

Gene	Region	SNP	Allele Change	T2DM-Related Traits	Dietary Factors	References
PPARG	3p25.2	rs1801282	C > G	HOMA-IR index	PUFA intake	[51,52,53]
TCF7L2	10q25.3	rs12573128	A > G	HOMA-IR index Oral glucose tolerance test	Fat intake	[54]
		rs12255372	G > T	T2DM risk	Carbohydrate intake	[55]
FTO	16q12.2	rs9939609	A > T	T2DM risk	Adherence to Mediterranean Diet	[56,57]
SLC30A8	8q24.11	rs11558471	A > G	Fasting glucose levels	Zinc intake Magnesium intake	[58,59]
		rs13266634	C > T	T2DM risk	*Trans*- and *cis*-beta-carotene and gamma-tocoferol intake	[60]
TRPM6	9q21.13	rs2274924	C > T	Fasting glucose levels	Magnesium intake	[59]
AS3MT	10q14.32	rs3740393	G > C	Fasting glucose levels	Magnesium intake	[59]
IRS1	2q36.3	rs2943641	C > T	HOMA-IR index	Vitamin D	[61]
GCKR	2p23	rs780094	C > T	Fasting insulin levels	Whole-grain intake	[62]
ADIPOQ *	3q27	SNP276 G > T	G > T	Fasting glucose levels	Carbohydrate intake	[63,64,65,66]
		SNP45 G > T	G > T	T2DM lower risk	Omega-3 intake	[67]
FABP2	4q28.31	Ala54Thr polymorphism	G > A	HOMA-IR index	SFA intake	[68]
CAV2	7q31.1	rs2270188	C > T	T2DM risk	SFA intake	[69]
PLIN	15q26.1	11482 G > A	G > A	HOMA-IR index	SFA fat and carbohydrates intake	[70]
		14995 A > T	A > T	HOMA-IR index	SFA fat and carbohydrates intake	[70]
CEBPA	19q13.1	rs12691	C > T	Oral glucose tolerance test HOMA-IR index	Fat intake	[71]
CLOCK	4q12	rs1801260	T > C	Fasting insulin levels HOMA-IR index QUICKI index	Fat and MUFA intake	[72]
CRY1	12q24.1	Rs2287161	G > C	Fasting insulin levels HOMA-IR index QUICKI index	Carbohydrate intake	[73]
SIRT1	10q21.3	rs7895833	A > G	Oral glucose tolerance test	Famine in prenatal life	[74]
		rs1467568	A > G	Oral glucose tolerance test	Famine in prenatal life	[74]

* Adiponectin (ADIPOQ).

### 3.1. Most Relevant T2DM Susceptibility Genes

Gene and environment interaction studies have shown a nice association between variants in peroxisome proliferator-activated receptor gamma (PPARG), TCF7L2 and fat mass and obesity-associated protein (FTO) genes, a Western dietary pattern and T2DM.

Interestingly, a proline to alanine substitution (Pro12Ala, rs1801282) in PPARG has been implicated in T2DM. The less frequent PPARG Ala12 variant reduces the risk of T2DM and is positively associated with insulin sensitivity [51]. Specific dietary factors, such as unsaturated fatty acids, which bind and upregulate PPARG, have been studied for gene and environment interactions [75]. In particular, some studies indicate Ala12 carriers may be more responsive to the beneficial effects of unsaturated fat and less sensitive to the adverse effects of total and saturated fat on glucose homeostasis compared to Pro12 homozygotes [52,53] (Table 2).

In the case of TCF7L2, it has been shown that diets with a low glycemic load reduce the risk of T2DM conferred by TCF7L2 [55,76]. In addition, TCF7L2 risk variant carriers may reduce their susceptibility to T2DM through dietary modifications, although this may require a much more intensive dietary regimen compared to non-risk carriers [77,78,79]. Moreover, the interaction between TCF7L2 rs12573128 and dietary fat intake was shown to influence insulin sensitivity and glucose tolerance [54]. Finally, in the Nurses’ Health Study, 1114 cases with T2DM and 1915 controls were genotyped for TCF7L2 (rs12255372), and dietary intake was assessed with a semi-quantitative food frequency questionnaire. The results demonstrated that carbohydrate quality and quantity modified the risk of T2DM, which indicates that changes in risk attributable to the TCF7L2 variant are increased under conditions of higher insulin demand [55] (Table 2).

The FTO gene has been consistently associated with obesity risk. However, the association between obesity risk alleles with T2DM remains controversial. A recent study aimed to determine whether these associations could be modulated according to the level of adherence to the Mediterranean diet. In this regard, a case-control study including 3430 T2DM cases and 3622 non-diabetic subjects, with no differences in body mass index (BMI), was performed. This study identified consistent gene-diet interactions with adherence to the Mediterranean diet for the FTO-rs9939609 variant. In addition, when adherence to the Mediterranean diet was low, carriers of the variant alleles showed a higher T2DM risk. In contrast, when adherence to the Mediterranean diet was high, these associations disappeared [56]. Another study demonstrated that patients with T2DM, who were carriers of the AA genotype of FTO rs9939609, showed increased fat and decreased fiber consumption, independent of BMI [57] (Table 2).

### 3.2. Other T2DM Susceptibility Genes

There are other studies that have established gene and dietetic interactions related to T2DM (Table 2). One of these genes is solute carrier family 30 (zinc transporter), member 8 (SLC30A8). The protein encoded by this gene is a zinc efflux transporter involved in the accumulation of zinc in intracellular vesicles. This gene is expressed at a high level only in the pancreas, particularly in the islets of Langerhans. The encoded protein colocalizes with insulin in the secretory pathway granules of insulin-secreting INS-1 cells [80]. In a 14-cohort meta-analysis that assessed the interaction of 20 genetic variants known to be related to glycemic traits and zinc metabolism with dietary zinc intake, as well as a 5-cohort meta-analysis that assessed the interaction with total zinc intake on fasting glucose levels in individuals of European ancestry without diabetes, a significant association of total zinc intake with lower fasting glucose levels was identified. However, the association with dietary zinc intake was not significant. Thus, a nominally significant, yet plausible, interaction between total zinc intake and the SLC30A8 rs11558471 variant on fasting glucose levels was observed. This result suggests a stronger inverse association between total zinc intake and fasting glucose in carriers of the fasting glucose-raising allele compared with non-carriers [58]. Furthermore, in a case-control study of 1796 participants (218 newly diagnosed with impaired glucose regulation, 785 newly diagnosed with T2DM and 793 with normal glucose tolerance test), the C allele of SLC30A8 rs13266634 was associated with higher odds of T2DM, whereas higher plasma zinc was associated with lower odds. Moreover, the inverse association of plasma zinc concentrations with T2DM was modified by SLC30A8 rs13266634 [81]. In a large-scale interaction study of 15 reports from the CHARGE (Cohorts for Heart and Aging Research in Genomic Epidemiology) Consortium, which included data from up to 52,684 participants of European descent without known diabetes, cross-sectional associations of dietary magnesium intake with fasting glucose and insulin and the interactions between magnesium intake and SNPs related to fasting glucose, insulin or magnesium on fasting glucose and insulin were analyzed. In this study, it was determined that rs11558471 in SLC30A8 showed a nominal interaction with magnesium consumption and fasting glucose [59]. It was also observed that magnesium consumption had a nominal interaction with rs2274924 in the magnesium transporter-encoding transient receptor potential cation channel, subfamily M, member 6 (TRPM6) and with arsenic (+3 oxidation state) methyltransferase (AS3MT) rs3740393 near cyclin M2 (CNNM2) related to fasting glucose [59] (Table 2). A significant interaction between SLC30A8 rs13266634 and three nutrients (*trans*- and *cis*-beta-carotene and gamma-tocopherol) was also identified [60]. Another study also explored the relationship between the insulin receptor substrate 1 (IRS1) variant rs2943641 and circulating levels of 25-hydroxyvitamin D (25(OH)D). Women homozygous for the minor allele rs2943641T with higher circulating 25(OH)D showed a lower risk of insulin resistance and T2DM compared to carriers of the major allele (rs2943641C) [61] (Table 2). Gene-whole grain intake interactions have also been established for the glucokinase regulatory protein (GCKR) variant rs780094 in a meta-analysis of 14 cohort studies; this study demonstrated that dietary whole grain intake potentially interacts with the cited variant. In subjects with the insulin-raising allele of rs780094, greater whole grain intake was associated with a smaller reduction of fasting insulin compared to individuals with the non-risk allele [62] (Table 2). Interactions of variants of the gene adiponectin with carbohydrate intake have also been explored [63]. In oriental individuals, the T allele of common adiponectin SNP276 G > T and SNP45 G > T has been associated with T2DM in Japanese individuals [64], whereas only SNP276 G > T has been associated with T2DM in Taiwanese patients [65]; the G allele of both SNPs has been associated with several components of the metabolic syndrome in non-obese and non-diabetic Korean men [66] (Table 2). Therefore, the difference in susceptibility of this SNP may be the result of different environmental factors, such as diet. Significant dose-response interactions were identified between the SNP276 G > T polymorphism and the dietary intake of carbohydrate. This previous study demonstrated the gene-nutrient interactions between the SNP276 G > T polymorphism and the level of carbohydrate intake modulated plasma fasting blood glucose and glycosylated hemoglobin (HbA1C) [63]. Moreover, adiponectin SNP45 G > T individuals who had a high intake of *n*-3 polyunsaturated fatty acids (PUFAs) showed a decreased risk of T2DM [66,67] (Table 2). Intestinal fatty acid-binding protein 2 (FABP2) Ala54Thr polymorphism, fat intake and insulin sensitivity have also been studied, and it was shown that insulin sensitivity was decreased in subjects with the Thr54 allele of the FABP2 polymorphism when saturated fatty acids (SFAs) were replaced by monounsaturated fatty acids (MUFAs) and carbohydrates [68] (Table 2). Finally, SNP-nutrient interaction effects between genes that encode fatty acid metabolism and lipid mobilization and dietary fat together with carbohydrate intake have been evaluated with respect to the risk of developing T2DM. In this regard, a significant relationship between the caveolin-2 (CAV2) rs2270188 TT genotype and fat and SAF intake with respect to T2DM has been observed [69] (Table 2). A significant gene-diet interaction between the perilipin (PLIN) 11482 G > A and PLIN 14995 A > T polymorphisms and dietary fat and carbohydrate intake has also been identified in the determination of insulin resistance in women. Of note, these gene-fat interactions were observed only for SFAs, but not for MUFAs or PUFAs [70] (Table 2). Another gene-environment interaction that has been studied is the CCAAT/enhancer-binding protein alpha (CEBPA) rs12691 SNP and 6 diabetes-related traits (fasting glucose and insulin levels, disposition index, insulin sensitivity index, HOMA-insulin resistance (HOMA-IR) index and acute insulin response to glucose) following 12 weeks of 4 dietary interventions (*i.e.*, high SFA diet, high MUFA diet, low-fat diet and low-fat-high-*n*-3 PUFA diet). The authors demonstrated that carriers of the minor A allele displayed impaired glucose metabolism measured by the disposition index, acute insulin response to glucose, insulin sensitivity index and HOMA-IR index compared with the G/G homozygotes [71] (Table 2).

### 3.3. CLOCK Gene Variants Linked to Diabetes

Dysregulation and genetic variations at the Circadian Locomotor Output Cycles Kaput (CLOCK) genes, which are responsible for the circadian system, have been associated with T2DM [82]. Two studies analyzed the gene-nutrient interaction between several CLOCK gene variants and diabetes-related traits. In this regard, one study focused on the associations between SNPs rs1801260, rs3749474 and rs4580704 with 3 diabetes-related traits (*i.e.*, insulin concentration, HOMA-IR index and quantitative insulin sensitivity check (QUICKI) index) following one year of dietary intervention (*i.e.*, 35% fat, 22% MUFAs *vs.* 28% fat, 12% MUFAs). The authors reported significant gene-diet interactions between rs1801260 SNP (for the major allele TT) following one year of a low-fat intervention. Subjects homozygous for TT displayed lower plasma insulin concentrations and HOMA-IR index and a higher QUICKI index [72] (Table 2). The other study analyzed the interaction between the cryptochrome 1 (Photolyase-Like) (CRY1) rs2287161 SNP and 4 diabetes-related traits (fasting glucose and insulin, HOMA-IR index and QUICKI index). This study found that individuals homozygous for the minor C allele had an increased carbohydrate intake (% of energy intake) that was associated with a significant increase in HOMA-IR index and fasting insulin, as well as a decrease in QUICKI [73] (Table 2).

### 3.4. Importance of Genotype by Macronutrient Interactions for T2DM-Related Traits

Recently, using genome-wide complex trait analysis, the genome-environment contribution of 14 dietary factors (glycemic load, total energy, protein, total fat, SFA, MUFA, PUFA, *n*-3 PUFA, *n*-6 PUFA, *n*-3:*n*-6 PUFA, carbohydrate, alcohol intake, trans fat and fiber) to the total phenotypic variance of 4 T2DM-related traits (fasting glucose, fasting insulin, HOMA-IR and HOMA of β cell function) were analyzed [83]. This study showed that for insulin and HOMA-IR, significant genome-nutrient variance contributions of carbohydrate were observed. In fact, 25.1% and 24.2% of the heritability of fasting insulin and HOMA-IR, respectively, could be explained by the genome-environment interaction of carbohydrate intake with the whole genome. However, the heritability explained by the genome alone for fasting insulin and HOMA-IR were 20.2% and 20.9%, respectively. In addition, for HOMA of β cell function, *n*-6 PUFA significantly contributed to the genome-nutrient interactions. In this regard, 39.0% of the heritability of HOMA of β cell function could be explained by the genome-environment interaction of *n*-6 PUFA with the genome, while the heritability explained by the main effect of the genome without these interactions was 18.7% for HOMA of β cell function [83].

Finally, genome-nutrient interactions during prenatal life can influence T2DM risk later in life. Indeed, it has been demonstrated that sirtuin (SIRT) 1 variants (rs7895833 and rs1467568) and prenatal exposure to famine significantly increased T2DM risk [74] (Table 2).

Thus, studies performed during the last decade have provided strong evidence to support a diet-genome interaction as an important factor leading to the development of T2DM.

## 4. The Effects of Nutrients on Gene Expression: Their Importance in DM

The “omics” technologies have been extensively used in an attempt to define molecular events involved in the health effects of nutrients. These technologies provide the opportunity to identify novel gene, protein and nutrient interactions. However, although the application of these technologies has become more accessible, they generate a large amount of complex data, as a specific nutrient could theoretically interact with all genes in the human genome.

**Figure 1:**
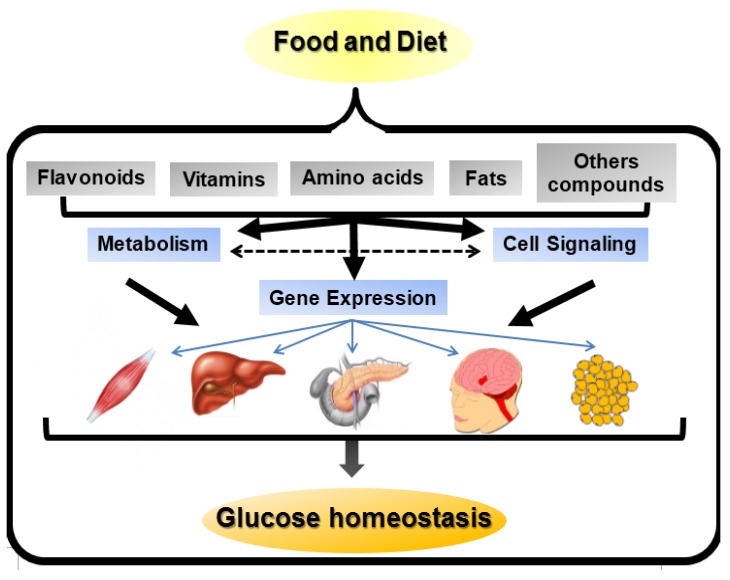
Nutrient-gene expression mechanisms. Nutrients present in food and diet can affect gene expression in a number of ways. They may directly act as ligands for transcription factors and change gene expression. Nutrients may be metabolized by different pathways, thereby modifying the concentration of substrates or intermediates that affect gene expression. Alternatively, the substrates or intermediates may act on or alter cell signaling pathways involved in gene expression. Moreover, nutrients may directly alter signal transduction pathways responsible for modifications in gene expression. Finally, the modifications in the signaling pathways, caused by nutrients, may modulate the metabolism of nutrients affecting gene expression. The modifications in gene expression may affect muscle, liver, pancreatic β cells, hypothalamus and adipose tissue, thereby regulating glucose homeostasis. The effects of these nutrient-gene interactions can be deleterious increasing DM risk and illness progression and complications or protective doing the opposite effects.

It was previously reported that food intake is a key component that affects the incidence of DM. Thus, the identification and analysis of nutrient/gene interactions are necessary steps to understand DM etiopathogenesis. In general, nutrients can affect gene expression via different mechanisms: (i) directly; (ii) through their metabolites and (iii) through signal transduction molecules (Figure 1).

### 4.1. Flavonoids-Gene Interactions in DM Pathogenesis

Numerous studies employing cell culture and living experimental animals support a beneficial effect of dietary flavonoids on glucose homeostasis [84]. Moreover, human studies have indicated that higher consumption of anthocyanins, particularly from blueberries, apples and pears, was consistently associated with a lower risk of DM [85]. These compounds were shown to regulate carbohydrate digestion, insulin secretion, insulin signaling and glucose uptake in insulin-sensitive tissues through various intracellular signaling pathways [86].

Interestingly, flavan-3-ols are present in many fruits, teas, cocoa and chocolate, especially epigallocatechin gallate (EGCG), and have been shown to improve insulin secretory function and the viability of β cells under conditions of glucotoxicity. These effects were mediated, at least in part, through increased expression of insulin receptor (Ir) substrate-2 (Irs2), protein kinase B (Akt), the forkhead box protein O1 (Foxo1) and pancreatic duodenal homeobox1 (Pdx1) [87]. EGCG has also been shown to protect insulin-producing β cells from pro-inflammatory cytokine-induced cytotoxicity via the modulation of B Cell CLL/Lymphoma 2 (Bcl-2) expression [88]. In addition, EGCG supplementation at pharmacological doses (1% in diet) improved insulin secretion from pancreatic β cells and preserved islet morphology in obese db/db mice by reducing the expression levels of carnitine palmitoyltransferase 1 (L-Cpt-1) and the endoplasmic reticulum stress marker DNA-damage-inducible transcript 3 (Ddit3), as well as its downstream targets protein phosphatase 1, regulatory subunit 15A (Ppp1r15a) and cyclin-dependent kinase inhibitor 1A (Cdkn1a) [89]. Thus, collectively, EGCG functions in many different roles that are associated with beneficial effects on DM. These changes include improvements in insulin secretion, glucose uptake, insulin resistance, glucose tolerance, oxidative stress, inflammation and mitochondrial function. In this regard, EGCG functions through modifications in the expression of genes involved in multiple signaling pathways to exert beneficial effects in DM (Table 3).

**Table 3:** Nutrient- or dietary pattern-gene interactions in the development of DM.

Nutrient	Gene Interaction	Function	Experimental Model	References
*Flavonoids*				
Epigallocatechingallate (EGCG)	↑Irs2, ↑Akt, ↑Foxo1, ↑Pdx1	↑Viability of β-cell, ↑insulin secretion	RIN-m5F cells	[87]
	↑Bcl-2	↓Apoptosis, ↑glucose uptake	RINm5F cells	[88]
	↓L-Cpt-1, ↓Ddit3, ↓Ppp1r15a, ↓Cdkn1a	↑Insulin secretion, preserve islet structure	db/db mice	[89]
Naringin or hesperidin	↑Gk (liver), ↑Glut4 (WAT), ↑Pparγ	↓Hyperglycemia	db/db mice	[90]
Naringin	↑Pparγ, ↑Hsp	↓Hyperglycemia, ↓hyperinsulinemia, ↓insulin resistance, ↑β cell function	HFD-STZ-induced T2DM rats	[91]
Anthocyanins	↑Glut4 (WAT, muscle), ↑Pparα, ↑Aco, ↑L-Cpt-1	↓Hyperglycemia, ↑insulin sensitivity	T2DM mice	[92]
	↓Lipogenic genes	↓Hyperglycemia, ↓hyperinsulinemia	HFD-DM mice	[93]
Quercetin	↓Cdkn1a	↓Hyperglycemia, ↑insulin plasma levels, ↑pancreatic cell proliferation	STZ-induced DM mice	[94]
Luteolin, apigenin	↓iNos	↓Apoptosis	RINm5F cells	[95]
Genistein	↑Ccnd1, ↓iNos	↓Hyperglycemia, ↑glucose tolerance, ↑insulin plasma levels	STZ-induced DM mice	[96]
*Another bioactive compounds*					
Oleanolic acid	↑Antioxidant enzymes genes, ↑phase II detoxification enzymes genes, ↓NF-κB	↑β-cell survival	Pancreatic islets	[97]
Berberine	↑Cyp7a1, ↑Igfbp1, ↑cell cycle genes, ↑NADPH metabolism genes	↓Fasting glucose, ↓insulin resistance	Diabetic Zucker rats	[98]
	↑CuZn-superoxide dismutase	↓Hyperglycemia	STZ-nicotinamide diabetic mice	[99]
*Vitamins*				
Vitamin D	↓Islet cytokine and chemokine genes	↓Insulitis	NOD mice	[100,101]
Biotin	↑Foxa2, ↑Pdx-1, ↑Hnf-4α, ↑Ins, ↑Gk, ↑Cacna1d, ↑Acac	↑Insulin secretion, ↑islet function	Mice	[102]
Riboflavin	↑IL-6	↓Cytokines-induced inflammation	NIT-1 cells	[103]
Nicotinamide	↑MafA	↑Insulin synthesis	INS-1 cells, pancreatic islets	[104]
*Amino acids*				
Leucine	↑mTor	↑Growth and proliferation	Pancreatic islets	[105]
Taurine	↑Pdx1, ↑Sur-1, ↑Gk, ↑Glut-2, ↑Ins	↑Insulin secretion, ↑insulin synthesis	OF1 mice, pancreatic islets culture	[106]
l-glutamine	↑Pdx1, ↑Calcineurin, ↑Acac	↑Insulin secretion, ↑proliferation	BRIN-BD11 beta-cells	[107]
*Dietary fats*				
Palmitate	↓Ins	↓Insulin secretion	Pancreatic islets	[108]
	↓Pdx-1, ↓MafA	↓Insulin secretion		[109]
HFD	↓Gpx1	↓Antioxidant defenses of β-cells	C57BL/6J mice	[110]
	↑Growth and development, ↑oxidative metabolism, ↑insulin processing and secretion, ↑signaling, ↑redox status	T2DM	NZO-mice	[111]
CHFD plus HFD	↓Pdx-1, ↓MafA, ↓Nkx6.1	↓Insulin secretion, ↓insulin synthesis, ↓β-cell survival	NZO-mice	[112]
Lipoic acid	↑Frk, ↑Gk, ↑G6pc2, ↑Phox	Protection against T2DM	High fructose-fed Wistar rats	[113]
Oleic acid	↓NPY, ↓AgRP	↓Food intake, ↓glucose production, ↓plasma glucose levels, ↓insulin plasma levels	Sprague-Dawley rats Sprague-Dawley or Zucker fatty rats	[114,115]

Naringin and hesperidin, the two major flavanones, are present in citrus fruits and have also been involved in protection against DM. For example, dietary supplementation with hesperidin or naringin (200 mg/kg) has been associated with anti-hyperglycemic effects in C57BL/KsJ-db/db mice following 5 weeks of treatment. This effect may primarily be due to an increase in liver glucokinase (Gk) and adipocyte glucose transporter type 4 (Glut4) [90]. In these mice, naringin and hesperidin treatment also led to the activation of the fat and liver peroxisome proliferator activated receptor (Ppar) γ [90] (Table 3). In T2DM rats fed a high-fat diet (HFD) and administered low-dose streptozotocin (STZ) injection, naringin was shown to dose-dependently ameliorate hyperglycemia, hyperinsulinemia and insulin resistance and improve β cell function. These effects were associated with increased expression of Pparγ and heat shock proteins (Hsp) in the livers of diabetic rats [91] (Table 3). However, to date, there remains a lack of data from clinical studies to support the anti-diabetic potential of these flavanones.

In the case of anthocyanidins, the observed anti-diabetic action appears to extend beyond their antioxidant property. Bilberry anthocyanins improved hyperglycemia and insulin sensitivity in T2DM mice by downregulating the expression of gluconeogenic enzymes, upregulating the expression of Pparα, L-Cpt-1, Glut4 and aconitase (Aco) in the livers of bilberry-supplemented T2DM mice, as well as upregulating the expression of Glut4 in the white adipose tissue (WAT) of bilberry-supplemented T2DM mice [92]. Moreover, this anthocyanin was shown to downregulate the expression of retinol-binding protein 4 (Rbp4) in the visceral fat [90] (Table 3). Finally, other anthocyanidins are known to control improvements in glucose homeostasis in high-fat-diet T2DM mice by downregulating lipogenic gene expression [93] (Table 3).

The most abundant flavonoids are flavonols, which are dispersed throughout plant-based foods. One of the most important dietary flavonols is quercetin. In STZ-induced DM mice, dietary supplementation with quercetin (0.5% in the diet for 2 weeks) lowered blood glucose and enhanced serum insulin concentrations. These effects were associated with the downregulation of genes associated with cell proliferation (Cdkn1a) in the liver and pancreas [94] (Table 3).

The major dietary flavones are apigenin and luteolin, which are found in celery, parsley and many herbs. In RIN cells, apigenin and luteolin treatment protected these cells from cytokine-induced apoptosis through the inhibition of inducible nitric oxide synthase (iNos) expression [95] (Table 3). However, it is not clear whether this effect also occurs in the islets *in vivo*.

The major dietary isoflavones are daidzein and genistein, which are primarily present in soy foods. In STZ-induced diabetic mice, it has been shown that genistein improved hyperglycemia, glucose tolerance and circulating insulin concentrations by increasing islet β cell proliferation, β cell mass and survival. These effects were a result of genistein-induced cyclin D1 (Ccnd1) expression, a major cell-cycle regulator required for growth in β cells, and reduced iNos expression [96] (Table 3).

Together, these studies indicate that dietary flavonoids exert their anti-diabetic effects by regulating the expression of different genes involved in various cellular signaling pathways in the pancreas, liver, skeletal muscle and WAT. These genes regulate nutrient-induced insulin release, insulin sensitivity and β cell proliferation and survival.

### 4.2. Bioactive Compounds-Gene Interactions in DM Pathogenesis

In addition to flavonoids, other bioactive compounds possess antidiabetic potential, such as triterpenoids. One of the most widely studied compounds of this family is oleanolic acid [97]. This compound is present in more than 120 plants and is especially abundant in the olive leaf [116,117]. Oleanolic acid has been demonstrated to improve insulin response and preserve the functionality and survival of pancreatic β cells. These actions appear to be derived from its interaction with transduction pathways that modulate the expression of key defensive genes, in which nuclear factor (erythroid-derived 2)-like 2 (Nfe2l2 or Nrf2) plays a very important role. In this regard, oleanolic acid is a potent inducer of the expression of antioxidant enzymes and other phase II detoxification enzymes, as wells as a repressor of the nuclear factor of the kappa light polypeptide gene enhancer in B-cells (Nf-κB) [97] (Table 3). Berberine is a quaternary ammonium salt of the protoberberine group of isoquinoline alkaloids and is the major active component of Rhizoma Coptidis. A meta-analysis study indicated that berberine appeared to generate antidiabetic effects via the reduction of hyperglycemia and dyslipidemia in T2DM [118]. In diabetic Zucker rats, berberine treatment has been shown to reduce fasting glucose and insulin resistance; in particular, the authors showed that berberine downregulated micro RNA 29-b (miR29-b) expression and upregulated a gene network involved in the cell cycle and intermediary and NADPH metabolism. Moreover, berberine normalized cytochrome P450, family 7, subfamily A, polypeptide 1 (Cyp7a1) and insulin-like growth factor binding protein 1 (Igfbp1) gene expression [98] (Table 3). Finally, in STZ-nicotinamide diabetic mice, berberine treatment lowered blood glucose levels, and these authors also demonstrated increased hepatic CuZn-superoxide dismutase expression [99] (Table 3).

### 4.3. Vitamins-Gene Interactions in DM Pathogenesis

Another group of nutrients with anti-diabetic properties are vitamins. For example, it has been demonstrated that vitamin D may improve β cell function by limiting chemokine expression, partially normalizing the expression of major histocompatibility complex (MHC) class I molecules and decreasing the density of MHC class I proteins on β cells [100] (Table 3). In addition, a very recent study demonstrated that vitamin D protects murine and human pancreatic islets against inflammation-induced β cell dysfunction and death. Vitamin D modifies the expression of approximately 250 genes, particularly genes related to functional groups involved in immune responses, chemotaxis, cell death and pancreatic β cell function/phenotype [101]. Low concentrations of a vitamin complex (ascorbic acid, β-carotene and α-tocopherol) were shown to reduce the expression of nicotinamide adenine dinucleotide phosphate (NADPH) oxidase subunits, superoxide dismutase and catalase genes in diabetic patients [119]. Moreover, eight weeks of biotin supplementation in mice increased the expression of forkhead box A2 (Foxa2), Pdx1, hepatocyte nuclear factor 4α (Hnf-4α), insulin (Ins), glucokinase (Gk), calcium channel, voltage-dependent, L type, alpha 1D subunit (Cacna1d) and acetyl-CoA carboxylase (Acac). These findings provide evidence for how biotin enhances insulin secretion and the expression of genes that favor islet function [102] (Table 3). In an insulinoma cell line (NIT-1) and murine islets, riboflavin treatment prevented the cytokine-induced increase in IL-6 mRNA expression [103] (Table 3). Nicotinamide also induced insulin gene expression in INS1-1 β cells via an increase in v-maf avian musculoaponeurotic fibrosarcoma oncogene homolog A (MafA) gene transcription [104] (Table 3).

### 4.4. Amino Acids-Gene Interaction in DM Pathogenesis

Amino acids are capable of direct modulation of insulin secretion and/or contribution to the maintenance of β cell function, which results in improved insulin release [120]. Amino acids, in addition to their effects on insulin secretion, may influence gene and protein expression in pancreatic islets (Table 3). For example, amino acid supplementation with taurine or leucine in control and malnourished mice increased the expression of genes and proteins essential for the insulin secretory process [120]. Leucine is the most effective amino acid in activating the mechanistic target of rapamycin (serine/threonine kinase) (mTor) complex [105]. As mTor is a key regulator of cell growth and proliferation [121], its activation is important in conditions of elevated demand for insulin, such as insulin resistance. Moreover, several studies have shown that branched-chain amino acids play an important role in the regulation of protein synthesis via the activation of mTOR in pancreatic β cells [122]. Taurine is a conditionally essential amino acid in humans that is involved in the control of glucose homeostasis; this effect was shown to be a result of increased insulin, sulfonylurea receptor-1 (Sur-1), Gk, glucose transporter type 2 (Glut-2), proconvertase and Pdx1 gene expression [106]. In the case of l-alanine and l-glutamine, dependent regulation of the expression of genes related to β cell signal transduction, metabolism and apoptosis has been observed, including some key metabolic genes, including ATP citrate lyase and catalase [123]. Moreover, l-glutamine strongly upregulates calcium binding proteins (calcineurin) [107]; this upregulation is important because calcineurin is a key activator of the nuclear factor of activated T cells (Nfat) in pancreatic β cells [124], and Nfat activation promotes β cell proliferation and the expression of metabolic enzyme genes [107]. Finally, l-glutamine induces dependent upregulation of Pdx1 and Acac expression [107].

### 4.5. Dietary Fat-Gene Interactions and Their Role in T2DM

Dietary fats also have important effects on gene expression in pancreatic β cells (Table 3). It is well known that consumption of a HFD is associated with an increased risk of T2DM [125]. However, the effects of fat on gene expression are varied. Following chronic exposure of β cells to palmitate, the inhibition of glucose-induced expression of prepro-insulin, as well as important transcription factors such as Pdx1 and MafA, caused β cell failure [108,109]. In addition, C57BL/6J mice fed a HFD (58% of calories from fat) showed a downregulation of glutathione peroxidase gene (Gpx1), which has been implicated in the antioxidant defenses of β cells [110]. Interestingly, Gpx1 regulates the expression of MafA, which is important in the regulation of insulin expression [110]. Thus, HFD-induced decreased expression of Gpx1 may be important in the pathogenesis of T2DM. An intriguing issue is that different mouse strains have distinct responses to a HFD diet when considering pancreatic β cell adaptation and insulin resistance. To investigate the role of different gene variants on these differences, the gene expression profile between C57BL/6J and AKR/J mice, which are both prone to HFD-induced metabolic syndrome, were analyzed following 12 weeks of HFD intake. Islets from HFD-fed AKR/J mice showed 202 genes upregulated and 270 genes downregulated compared to HFD-fed C57BL/6j mice. A subsequent analysis indicated that the most profound differences observed were in genes related to secreted proteins, membrane receptors, extracellular matrix proteins and lipid metabolism [126]. An interesting study evaluated the diet-dependent genome-wide gene expression patterns of New Zeeland Obese (NZO) mice following a carbohydrate-free high fat-diet (CHFD) or a normal HFD [111]. NZO mice fed a HFD developed T2DM; however, when the mice were fed a CHFD, they did not develop T2DM [111]. In the islets of HFD- and CHFD-fed mice, 2109 genes were differentially expressed, showing changed of at least 1.5-fold. In response to a HFD, 1496 genes were upregulated and 613 genes were downregulated. In the HFD group, gene expression changes were associated with growth and development, such as cyclin-dependent kinase inhibitor 1b (Cdkn1b), purine rich element binding protein A (Pura), dachshund 1 (Dach1), tetraspanin 8 (Tspan 8) and paired box gene 6 (Pax6). Genes involved in insulin production and secretion, such as proprotein convertase subtilisin/kexin type 1 (Pcsk1), proprotein convertase subtilisin/kexin type 2 (Pcsk2), ribosomal protein S27-like (Rps27l), proteasome subunit beta type 7 (Psmb7), vesicle-associated membrane protein 8 and 5 (Vamp8 and Vamp5) and vesicle transport through interaction with *t*-SNAREs 1B homologue (Vti1b), were also differentially regulated. In addition, genes involved in the cellular redox state, such as thioredoxin interactin protein (Txnip), catalase (Cat), Gpx1 and peroxiredoxin 4 and 5 (Prdx4 and Prdx5), were also increased in the islets of HFD animals [126]. Moreover, genes related to signaling were also upregulated, which primarily included cholecystokinin (Cck), RAS-homologue enriched in brain (Rheb) and dual specificity phosphatase 6 (Dusp6); these changes represented a greater than 4-fold increase. Finally, regarding genes related to metabolism, genes with a larger than 4-fold upregulation included GDP-mannose 4, 6-dehydrate (Gmds), NADH dehydrogenase (ubiquinone), 1 alpha subcomplex 1 (Ndufa1), squalene epoxidase (Sqle), ATP synthase H^+^ transporting mitochondrial F1 complex beta subunit (Atp5b), phosphoribosyl pyrophosphate synthetase 1 (Prps1), succinate-CoA ligase GDP-forming alpha subunit (Suclg1) and paraoxonase 3 (Pon3). In the case of the liver, HFD-fed mice showed an upregulation of carbohydrate-responsive element-binding protein (Chrebp), pyruvate kinase liver and RBC (Pklr) and stearoyl-CoA desaturase (Scd1) [111]. In this study, the islets from mice that received a HFD or CHFD showed enhanced oxidative metabolism because the genes for the oxidative phosphorylation (OXPHOS) metabolic pathway were upregulated, which suggests an increased flux of fatty acid metabolism through mitochondrial oxidation, specifically in HFD-fed mice. Because OXPHOS is a source of reactive oxygen species (ROS), the increased formation of reactive oxygen species (ROS) can produce mitochondrial and β cell apoptosis. Indeed, the involvement of oxidative stress has been proposed in the lipotoxicity hypothesis of T2DM [127]. Furthermore, the gene expression of cellular redox state regulators was also upregulated in HFD-fed mice. In another study, NZO mice were first fed with CHFD for 18 weeks and then fed with HFD. Two days after the HFD, the islets showed decreased expression of Pdx1, MafA and NK6 homeobox 1 (Nkx6.1) [112], which are transcription factors important for β cell integrity and survival.

Lipoic acid is an organosulfur compound that is derived from octanoid acid. Lipoic acid is a potent lipophilic free radical scavenger with antioxidant effects, which has been shown to decrease blood glucose levels and glycosylated hemoglobin (HbA1c) in T2DM patients [128]. In addition, this compound was shown to prevent the increased fructokinase (Frk), Gk, glucose-6-phosphatase (G6pc2) and p22 (Phox) gene expression observed in fructose-fed rats. Thus, the authors suggested that lipoic acid could prevent the transition from impaired glucose tolerance to T2DM [113] (Table 3).

In the case of *in vitro* experiments with mouse and human islets cultured with high levels of palmitate, an alteration of genes involved in lipid metabolism, inflammation and oxidative stress has been observed [129,130,131].

### 4.6. Food-Gene Interactions in DM Pathogenesis: Human Studies

Substantially fewer studies have been performed in humans concerning nutrient-gene interactions in pancreatic β cell function- or insulin sensitivity-related genes. In one study, human *in vivo* gene expression changes in peripheral blood mononuclear cells following an acute ingestion of virgin olive oil were evaluated in 11 healthy volunteers. These data showed short time-course changes in the expression of genes related to insulin resistance, such as ADAM metallopeptidase domain 17 (ADAM17), adrenoreceptor beta 2 (ADRB2), lipoic acid synthetase (LIAS), arachidonate 5-lipoxygenase-activating protein (ALOX5AP), thrombospondin receptor (CD36), *O*-linked *N*-acetylglucosamine transferase (OGT) and PPARBP [132]. The effects of carbohydrates were also studied. In a parallel study design, 47 subjects with metabolic syndrome were fed two different types of carbohydrates (a rye pasta diet with a low postprandial insulin response and an oat-wheat-potato diet with high postprandial insulin response). In the rye pasta diet group, the downregulation of 71 genes was observed; some genes were related to insulin signaling, such as downregulation of the insulin receptor and insulin*-*like growth factor binding protein 5 (IGFBP-5) genes. On the other hand, in the oat-wheat-potato diet group, the upregulation of 62 genes linked to stress, cytokine-chemokine-mediated immunity and the interleukin pathway was observed [133]. Finally, studies on the effects of calorie restriction in obese men who lost 5% of their body weight showed a modification in the expression of 385 genes (158 upregulated and 227 downregulated) in peripheral blood mononuclear cells; some of these genes were related to insulin sensitivity [134].

**Figure 2:**
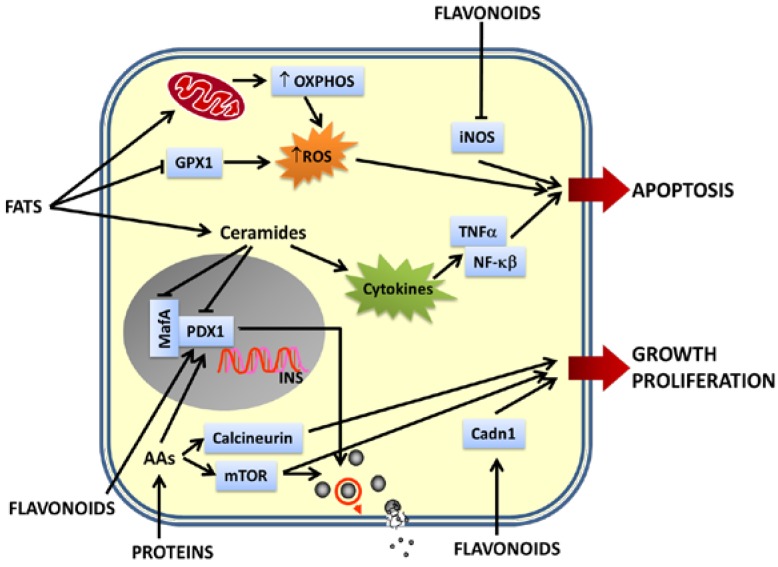
Effects of nutrients on β cell gene expression. Pancreatic β cells are able to sense dietary nutrients and respond to them releasing insulin. Different nutrients and their metabolites affect transcription of genes very important for maintenance of β cell function and integrity. Flavonoids upregulate the expression of genes involved in insulin synthesis, nutrient-induced insulin release and β cell proliferation and downregulate genes implicated in β cell apoptosis. Proteins positively regulate insulin synthesis, insulin release, β cell proliferation and growth upregulating the expression of mTOR, calcineurin and Pdx1. Fats upregulate OXPHOS genes leading to the generation of metabolic coupling factors critical for insulin exocytosis. On the other hand, a chronic exposure of β-cells to high levels of fats (mainly saturated fatty acids) induces excessive levels of ROS and pro-inflammatory cytokines, leading to an increased apoptosis. The upregulation of the expression of cytokine genes and genes involved in pro-inflammatory signaling pathways, together with the downregulation of genes implicated in the antioxidant defenses of β cells, contribute to β cell apoptosis. Moreover, chronic exposure to fats and their byproducts downregulate the expression of genes necessary for insulin synthesis, nutrient-induced insulin release, β cell integrity, maintenance and survival (Pdx1 and MafA). Impairment of β-cell function is a hallmark of pancreatic β-cell failure and may lead to development of DM.

All these studies show that gene expression, in pancreatic islets, is very sensitive to nutrients and bioactive compounds present in food. The altered expression of genes involved in β cell nutrient sensing, insulin synthesis, cell cycle, survival/apoptosis and cell maintenance can impair β cell function and at the end facilitates β cell failure (Figure 2).

### 4.7. Nutrient-Gene Interactions in Hypothalamus Are Also Involved in DM Pathogenesis

Another important issue to be considered is that to completely determine the etiopathogenesis of T2DM, it is necessary to understand the physiological regulation of energy homeostasis. In the end, this is governed by the hypothalamus. The role of hypothalamus in diabetes has already been demonstrated. Okamoto *et al.* [135] showed that restoration of insulin-signaling in the β cell and liver was unable to entirely reverse insulin resistance in a full body insulin receptor knockout mice. In addition, neuron-specific insulin receptor knockout mice exhibited a decreased response to insulin and hyperinsulinemia [136]. Thus, the hypothalamus has the capacity to sense and respond to nutrients, such as glucose and fatty acids. This results in lowering food intake and hepatic glucose production, to ultimately control metabolic homeostasis [137]. In this regard, different levels of glucose concentrations are able to increase the expression of the anorexigenic proopiomelanocortin (POMC) gene, in hypothalamic neuronal cell lines [138]. In addition, Obici *et al.* [114] demonstrated that fatty acid metabolization participates in hypothalamus glucose homeostasis regulation. For example, the intracerebroventricular administration of the MUFA oleic acid reduces the hypothalamic expression of neuropeptide Y (NPY) and agouti-related protein (AgRP) [114,115], which in turns lowers food intake and glucose production. This results in a decrease in plasma insulin and glucose levels. Thus, data generated in the past decade indicate that hypothalamus is a key regulator of glucose homeostasis by sensing nutrients such as fatty acids and glucose. These nutrients are able to modify the expression of genes involved in energy balance. The knowledge of the molecular mechanisms that link nutrient input and brain responses will help us to identify possible central targets to fight against T2DM.

## 5. Epigenetics, Micro RNAs (miRNAs) and Diet: Are They Involved in DM?

Previous epigenetic studies have focused on the heritable alteration of DNA and proteins, linking the DNA and histones, which induces modifications in chromatin structure without changing the nucleotide sequence. Modulations in gene expression can be caused by epigenetic mechanisms such as DNA methylation, histone modifications, small and non-coding RNAs [139]. Non-coding RNAs (ncRNAs) have been implicated in the epigenetic regulation of gene expression, and recent studies have shown that miRNAs can induce chromatin remodeling. miRNAs are single-stranded RNA molecules that range in size from 18 to 22 nucleotides. The mammalian genome encodes several hundred miRNAs that fine-tune gene expression through the modulation of target mRNAs [140]. These findings suggest that DNA methylation, histone modification and miRNAs may function in concert to regulate gene expression [141].

Various diet and dietary interventions have been associated with epigenetic changes that alter cellular signaling [142]. The first insights regarding the importance of nutritional status in epigenetic changes and showing that these epigenetic changes could have heritable health consequences on a long-term scale were obtained from the Dutch Famine Cohort [143]. Studies with this cohort have shown that serious nutritional deprivation during pregnancy caused an increased risk of metabolic disorders in the offspring several decades later [144]. This period of maternal starvation was very significant and caused marked differences in disease outcomes; the first trimester of pregnancy was particularly vulnerable to disease outcome in adulthood. The studies from the Dutch Famine Cohort have provided two principal lessons. First, there is a critical developmental time window where dietary pattern can induce epigenetic changes; second, these epigenetic changes are passed to offspring.

To better address these points, various animal models have been developed. For example, using HFD-T2DM male rats, the F1 female offspring showed reduced β cell area and insulin secretion, together with glucose intolerance, without changes in body weight [145]. The islets of the F1 female offspring showed differential expression of many genes involved in Ca^2+^, mitogen-activated protein kinase and Wnt signaling, apoptosis and cell cycle regulation [145]. Similarly, in pregnant C57BL6J mice, food deprivation resulted in β cell mass reduction and an increased risk of β cell failure in offspring [146].

DNA methylation profiling of human islets that originated from diabetic and non-diabetic donors showed DNA methylation in the promoter regions of 254 genes, where the vast majority of CpG sites were hypomethylated in islets from diabetic donors [147]. However, *in vitro* incubation of islets with 30 mM glucose for 48 h did not induce methylation of several CpG sites tested [147].

Diverse dietary components, such as amino acids, carbohydrates, fatty acids, vitamins and phytochemicals, have been found to affect the expression profile of miRNAs or their functions [142]. In addition, miRNAs have been shown to be involved in T2DM. For example, miRNAs play major roles in pancreatic islet development, β cell dysfunction, insulin synthesis and secretion and insulin resistance [148]. Studies based on miRNA microarray analysis have identified many different miRNAs involved in the pathology of both T1DM and T2DM; these miRNAs include miR-375, miR-29, miR-9, miR-124a, miR-195, miR-222, miR-126, miR-133a, miR-296, miR-96, miR-34a, miR-146b, miR-657, miR-30d, miR-103, miR-107, miR-1, miR-125, miR-27, miR-122, miR-320, miR-21 and miR-30a [148]. In addition, Let-7 family miRNAs are related to the insulin signaling pathway, glucose metabolism and insulin sensitivity [149]. Some miRNAs (Let-7 family, miR-27, miR-29, miR-103, miR-107 and miR-122) are also deregulated by polyphenols, such as EGCG, ellagitannin, resveratrol, genistein and quercetin [150]. In addition, miR-21 expression is altered in response to a HFD, PUFAS and caloric restriction [151], and miR-222 levels were shown to be increased following nutrient deprivation of folate and vitamin B [151]. The Let-7 family also exhibited decreased levels in the presence of fats, changes in the presence of glucose and increased levels in the presence of vitamin A derivatives and folate, methionine and choline deficiency [151].

## 6. Conclusions

T1DM involves the specific autoimmune destruction of pancreatic β cells. The single most important genetic determinant of T1DM susceptibility is the human leukocyte antigen (HLA). However, HLA only explains approximately 60% of the genetic influence of T1DM. In addition, other non-HLA genetic polymorphisms indicate that environmental cues, such as diet-related issues, contribute to this disease. One important challenge is to identify the specific food determinants that trigger β cell autoimmunity and the progression from persistent β cell autoimmunity to the clinical onset of illness. Apart from vitamin D, the early intake of cow’s milk and gluten, and the protective effects of breastfeeding, there is limited evidence that other nutrients may affect T1DM onset. In addition, the role of the gut microbiota in the etiology of T1DM has gained importance. It is also critical to understand the potential role of nutritional factors in the origin and progression of T1DM, as well as to identify polymorphisms that link dietary factors with the drivers of T1DM development. To progress in this direction, carefully well-designed longitudinal studies are needed to understand the relationships among nutrients, microbiota, polymorphisms, genetic susceptibility and autoimmunity. T2DM is multifactorial and arises from complex interactions between the genetic makeup and environment. To date, the underlying gene-nutrient interactions that cause T2DM are not completely understood. Because of the complex genetic picture of T2DM, the role of nutrients and dietary patterns in the etiopathogenesis of the disease and related traits will most likely be multifactorial at the molecular level. Certainly, different pathophysiological pathways involve nutrient-induced failure of β cells and insulin resistance. Moreover, newly identified factors, such as epigenetic modification and miRNAs, likely have a substantial role in the impact of nutrients in T2DM. The integration of an individual’s genetic predisposition, gene-nutrient interactions, epigenetic programing and the involvement of nutrients in this issue, together with the knowledge obtained at all “omic” levels, produce a complex puzzle; thus, a systems biology approach will be required to advance knowledge of the origin, progression, prevention and treatment of T2DM. This will imply a deeper characterization of patients at the omic and clinical levels, which includes as many nutrient and/or dietary patterns as possible and the integration of data in interaction networks. Furthermore, the intestinal microbiota should be considered, as well as food genomes. Another challenge will be to translate the large data set generated by the different omics approaches into concrete knowledge that could be useful in the discovery of early molecular events that occur in DM and the identification of better biomarkers and diagnostic tools. These factors should enable personalized approximations thorough tailored diets that may contribute to DM prevention, which is the biggest challenge in addressing the burden of DM.

## References

[1] Georgoulis M., Kontogianni M.D., Yiannakouris N. (2014). Mediterranean diet and diabetes: Prevention and treatment. Nutrients.

[2] International Diabetes Federation (2013). IDF Diabetes Atlas.

[3] Dib S.A., Gomes M.B. (2009). Etiopathogenesis of type 1 diabetes mellitus: Prognostic factors for the evolution of residual beta cell function. Diabetol. Metab. Syndr..

[4] Hansen L., Pedersen O. (2005). Genetics of type 2 diabetes mellitus: Status and perspectives. Diabetes Obes. Metab..

[5] Schulze M.B., Hu F.B. (2005). Primary prevention of diabetes: What can be done and how much can be prevented?. Annu. Rev. Public Health.

[6] Corella D., Ordovas J.M. (2009). Nutrigenomics in cardiovascular medicine. Circ. Cardiovasc. Genet..

[7] Brennan R.O. (1976). Nutrigenetics: New Concepts for Relieving Hypoglycemia.

[8] Corella D., Carrasco P., Sorlí J.V., Estruch R., Rico-Sanz J., Martínez-González M.Á., Salas-Salvadó J., Covas M.I., Coltell O., Arós F. (2013). Mediterranean diet reduces the adverse effect of the TCF7L2-rs7903146 polymorphism on cardiovascular risk factors and stroke incidence: A randomized controlled trial in a high-cardiovascular-risk population. Diabetes Care.

[9] Daar A.S., Singer P.A., Persad D.L., Pramming S.K., Matthews D.R., Beaglehole R., Bernstein A., Borysiewicz L.K., Colagiuri S., Ganguly N. (2007). Grand challenges in chronic non-communicable diseases. Nature.

[10] Scott J. (1987). Molecular genetics of common diseases. Br. Med. J..

[11] Sladek R., Rocheleau G., Rung J., Dina C., Shen L., Serre D., Boutin P., Vincent D., Belisle A., Hadjadj S. (2007). A genome-wide association study identifies novel risk loci for type 2 diabetes. Nature.

[12] Stratigopoulos G., Padilla S.L., LeDuc C.A., Watson E., Hattersley A.T., McCarthy M.I., Zeltser L.M., Chung W.K., Leibel R.L. (2008). Regulation of FTO/FTM gene expression in mice and humans. Am. J. Physiol. Regul. Integr. Comp. Physiol..

[13] Thorisson G.A., Stein L.D. (2003). The SNP Consortium website: Past, present and future. Nucleic Acid Res..

[14] Mitchell A.A., Chakravarti A., Cutler D.J. (2005). On the probability that a novel variant is a disease-causing mutation. Genome Res..

[15] Bailey-Wilson J.E., Wilson A.F. (2011). Linkage analysis in the next generation sequencing era. Hum. Hered..

[16] Zanda M., Onengut-Gumuscu S., Walker N., Shtir C., Gallo D., Wallace C., Smyth D., Todd J.A., Hurles M.E., Plagnol V. (2014). A genome-wide assessment of the role of untagged copy number variants in type 1 diabetes. PLoS Genet..

[17] Ziegler A.G., Nepom G.T. (2010). Prediction and pathogenesis in type 1 diabetes. Immunity.

[18] Virtanen S.M., Knip M. (2003). Nutritional risk predictors of beta-cell autoimmunity and type 1 diabetes at a young age. Am. J. Clin. Nutr..

[19] Knip M., Virtanen S.M., Becker D., Dupré J., Krischer J.P., Åkerblom H.K., TRIGR Study Group (2011). Early feeding risk of type 1 diabetes: Experiences from the Trial to Reduce Insulin-dependent diabetes mellitus in the Genetically at Risk (TRIGR). Am. J. Clin. Nutr..

[20] Norris J.M., Barriga K., Klingensmith G., Hoffman M., Eisenbarth G.S., Erlich H.A., Rewers M. (2003). Timing of initial cereal exposure in infancy and risk of islet autoimmunity. JAMA.

[21] Hyppönen E., Läärä E., Reunanen A., Järvelin M.R., Virtanen S.M. (2001). Intake of vitamin D and risk of type 1 diabetes: A birth-cohort study. Lancet.

[22] Sildorf S.M., Fredheim S., Svensson J., Buschard K. (2012). Remission without insulin therapy on gluten-free diet in a 6-year old boy with type 1 diabetes mellitus. BMJ Case Rep..

[23] Wu G.D., Chen J., Hoffmann C., Bittinger K., Chen Y.Y., Keilbaugh S.A., Bewtra M., Knights D., Walters W.A., Knight R. (2011). Linking long-term dietary patterns with gut microbial enterocytes. Science.

[24] Patrick C., Wang G.S., Lefebvre D.E., Crookshank J.A., Sonier B., Eberhard C., Mojibian M., Kennedy C.R., Brooks S.P., Kalmokoff M.L. (2013). Promotion of autoimmune diabetes by cereal diet in the presence or absence of microbes associated with gut immune activation, regulatory imbalance, and altered cathelicidin antimicrobial peptide. Diabetes.

[25] Bobbala D., Chen X.L., Leblanc C., Mayhue M., Stankova J., Tanaka T., Chen Y.G., Ilangumaran S., Ramanathan S. (2012). Interleukin-15 plays an essential role in the pathogenesis of autoimmune diabetes in the NOD mouse. Diabetologia.

[26] Sakaguchi S., Ono M., Setoguchi R., Yagi H., Hori S., Fehervari Z., Shimizu J., Takahashi T., Nomura T. (2006). Foxp3^+^ CD25^+^ CD4^+^ natural regulatory T cells in dominant self-tolerance and autoimmune disease. Immunol. Rev..

[27] Salmond R.J., Filby A., Qureshi I., Caserta S., Zamoyska R. (2009). T-cell receptor proximal signaling via the Src-family kinases, Lck and Fyn, influences T-cell activation, differentiation, and tolerance. Immunol. Rev..

[28] Nijnik A., Pistolic J., Wyatt A., Tam S., Hancock R.E. (2009). Human cathelicidin peptide LL-37 modulates the effects of IFN-gamma on APCs. J. Immunol..

[29] Phillips C.M. (2013). Nutrigenetics and metabolic disease: Current status and implications for personalized nutrition. Nutrients.

[30] Vimaleswaran K.S., Loos R.J. (2010). Progress in the genetics of common obesity and type 2 diabetes. Expert Rev. Mol. Med..

[31] Temelkova-Kurktschiev T., Stefanov T.S. (2012). Lifestyle and genetics in obesity and type 2 diabetes. Exp. Clin. Endocrinol. Diabetes.

[32] Kaput J., Noble J., Hatipoglu B., Kohrs K., Dawson K., Bartholomew A. (2007). Application of nutrigenomic concepts to type 2 diabetes mellitus. Nutr. Metab. Cardiovasc. Dis..

[33] Flint J., Valdar W., Shifman S., Mott R. (2005). Strategies for mapping and cloning quantitative trait genes in rodents. Nat. Rev. Genet..

[34] Kaput J., Dawson K. (2007). Complexity of type 2 diabetes mellitus data sets emerging from nutrigenomic research: A case for dimensionality reduction?. Mutat. Res..

[35] Hindorff L.A., MacArthur J., Morales J., Junkins H.A., Hall P.N., Klemm A.K., Manolio T.A. A Catalog of Published Genome-Wide Association Studies. http://www.genome.gov/gwastudies.

[36] Dupuis J., Langenberg C., Prokopenko I., Saxena R., Soranzo N., Jackson A.U., Wheeler E., Glazer N.L., Bouatia-Naji N., Gloyn A.L. (2010). New genetic loci implicated in fasting glucose homeostasis and their impact on type 2 diabetes risk. Nat. Genet..

[37] McCarthy M.I. (2010). Genomics, type 2 diabetes, and obesity. N. Engl. J. Med..

[38] Saxena R., Hivert M.F., Langenberg C., Tanaka T., Pankow J.S., Vollenweider P., Lyssenko V., Bouatia-Naji N., Dupuis J., Jackson A.U. (2010). Genetic variation in GIPR influences the glucose and insulin responses to an oral glucose challenge. Nat. Genet..

[39] Strawbridge R.J., Dupuis J., Prokopenko I., Barker A., Ahlqvist E., Rybin D., Petrie J.R., Travers M.E., Bouatia-Naji N., Dimas A.S. (2011). Genome-wide association identifies nine common variants associated with fasting proinsulin levels and provides new insights into the pathophysiology of type 2 diabetes. Diabetes.

[40] Florez J.C. (2008). Newly identified loci highlight beta cell dysfunction as a key cause of type 2 diabetes: Where are the insulin resistance genes?. Diabetologia.

[41] Grant S.F., Thorleifsson G., Reynisdottir I., Benediktsson R., Manolescu A., Sainz J., Helgason A., Stefansson H., Emilsson V., Helgadottir A. (2006). Variant of transcription factor 7-like 2 (TCF7L2) gene confers risk of type 2 diabetes. Nat. Genet..

[42] Zeggini E., McCarthy M.I. (2007). TCF7L2: The biggest story in diabetes genetics since HLA?. Diabetologia.

[43] Chandak G.R., Janipalli C.S., Bhaskar S., Kulkarni S.R., Mohankrishna P., Hattersley A.T., Frayling T.M., Yajnik C.S. (2007). Common variants in the TCF7L2 gene are strongly associated with type 2 diabetes mellitus in the Indian population. Diabetologia.

[44] Horikoshi M., Hara K., Ito C., Nagai R., Froguel P., Kadowaki T. (2007). A genetic variation of the transcription factor 7-like 2 gene is associated with risk of type 2 diabetes in the Japanese population. Diabetologia.

[45] Lehman D.M., Hunt K.J., Leach R.J., Hamlington J., Arya R., Abboud H.E., Duggirala R., Blangero J., Göring H.H., Stern M.P. (2007). Haplotypes of transcription factor 7-like 2 (TCF7L2) gene and its upstream region are associated with type 2 diabetes and age of onset in Mexican Americans. Diabetes.

[46] Helgason A., Pálsson S., Thorleifsson G., Grant S.F., Emilsson V., Gunnarsdottir S., Adeyemo A., Chen Y., Chen G., Reynisdottir I. (2007). Refining the impact of TCF7L2 gene variants on type 2 diabetes and adaptive evolution. Nat. Genet..

[47] Lyssenko V., Lupi R., Marchetti P., Del Guerra S., Orho-Melander M., Almgren P., Sjögren M., Ling C., Eriksson K.F., Lethagen A.L. (2007). Mechanisms by which common variants in the TCF7L2 gene increase the risk of type 2 diabetes. J. Clin. Investig..

[48] Cornelis M.C., Hu F.B. (2012). Gene-enviroment interactions in the development of type 2 diabetes: Recent progress and continuing challenges. Annu. Rev. Nutr..

[49] Lee Y.C., Lai C.Q., Ordovas J.M., Parnell L.D. (2011). A database of gene-enviroment interactions pertaining to blood lipid traits, cardiovascular disease and type 2 diabetes. J. Data Mining Genomics Proteomics.

[50] Harrington J.M., Phillips C.M. (2014). Nutrigenetics: Bridging two worlds to understand type 2 diabetes. Curr. Diabetes Rep..

[51] Gouda H.N., Sagoo G.S., Harding A.H., Yates J., Sandhu M.S., Higgins J.P. (2010). The association between the peroxisome proliferator-activated-receptor gamma 2 (PPARG2) Pro12Ala gene variant and type 2 diabetes mellitus: HuGe review and meta-analysis. Am. J. Epidemiol..

[52] Lamri A., Abi-Khalil C., Jaziri R., Velho G., Lantieri O., Vol S., Froguel P., Balkau B., Marre M., Fumeron F. (2012). Dietary fat intake and polymorphisms at the PPARG locus modulate BMI and type 2 diabetes risk in the D.E.S.I.R. prospective study. Int. J. Obes..

[53] Luan J., Browne P.O., Harding A.H., Halsall D.J., O’Rahilly S., Chatterjee V.K., Wareham N.J. (2001). Evidence for gene-nutrient interaction at the PPARgamma locus. Diabetes.

[54] Ruchat S.M., Elks C.E., Loos R.J., Vohl M.C., Weisnagel S.J., Rankinen T., Bouchard C., Pérusse L. (2009). Evidence of interaction between type 2 diabetes susceptibility genes and dietary fat intake for adiposity and glucose homeostasis-related phenotypes. J. Nutrigenet. Nutrigenomics.

[55] Cornelis M.C., Qi L., Kraft P., Hu F.B. (2009). TCF7L2, dietary carbohydrate, and risk of type 2 diabetes in US women. Am. J. Clin. Nutr..

[56] Ortega-Azorín C., Sorlí J.V., Asensio E.M., Coltell O., Martínez-González M.Á., Salas-Salvadó J., Covas M.I., Arós F., Lapetra J., Serra-Majem L. (2012). Associations of the FTO rs9939609 and the MC4R rs17782313 polymorphisms with type 2 diabetes are modulated by diet, being higher when adherence to the Mediterranean diet pattern is low. Cardiovasc. Diabetol..

[57] Steemburgo T., Azevedo M.J., Gross J.L., Milagro F.I., Campión J., Martínez J.A. (2013). The rs9939609 polymorphism in the FTO gene is associated with fat and fiber intakes in patients with type 2 diabetes. J. Nutrigenet. Nutrigenomics.

[58] Kanoni S., Nettleton J.A., Hivert M.F., Ye Z., van Rooij F.J., Shungin D., Sonestedt E., Ngwa J.S., Wojczynski M.K., Lemaitre R.N. (2011). Total zinc intake may modify the glucose-raising effect of a zinc transporter (SLC30A8) variant: A 14-cohort meta-analysis. Diabetes.

[59] Hruby A., Ngwa J.S., Renström F., Wojczynski M.K., Ganna A., Hallmans G., Houston D.K., Jacques P.F., Kanoni S., Lehtimäki T. (2013). Higher magnesium intake is associated with lower fasting glucose and insulin, with no evidence of interaction with select genetic loci, in a meta-analysis of 15 CHARGE Consortium Studies. J. Nutr..

[60] Patel C.J., Chen R., Kodama K., Ioannidis J.P.A., Butte A.J. (2013). Systematic identification of interactions effects between genome- and environment-wide associations in type 2 diabetes mellitus. Hum. Genet..

[61] Zheng J.S., Parnell L.D., Smith C.E., Lee Y.C., Jamal-Allial A., Ma Y., Li D., Tucker K.L., Ordovas J.M., Lai C.Q. (2014). Circulating 25-hydroxyvitamin D, IRS1 variant rs2943641, and insulin resistance: Replication of a gene-nutrient interaction in 4 populations of different ancestries. Clin. Chem..

[62] Nettleton J.A., McKeown N.M., Kanoni S., Lemaitre R.N., Hivert M.F., Ngwa J., van Rooij F.J., Sonestedt E., Wojczynski M.K., Ye Z. (2010). Interactions of dietary whole-grain intake with fasting glucose- and insulin-related genetic loci in individuals of European descent: A meta-analysis of 14 cohort studies. Diabetes Care.

[63] Hwang J.Y., Park J.E., Choi Y.J., Huh K.B., Chang N., Kim W.Y. (2013). Carbohydrate intake interacts with SNP276G > T polymorphism in the adiponectin gene to affect fasting blood glucose, HbA1C, and HDL cholesterol in Korean patients with type 2 diabetes. J. Am. Coll. Nutr..

[64] Hara K., Boutin P., Mori Y., Tobe K., Dina C., Yasuda K., Yamauchi T., Otabe S., Okada T., Eto K. (2002). Genetic variation in the gene encoding adiponectin is associated with an increased risk of type 2 diabetes in the Japanese population. Diabetes.

[65] Yang W.S., Yang Y.C., Chen C.L., Wu I.L., Lu J.Y., Lu F.H., Tai T.Y. (2007). Adiponectin SNP276 is associated with obesity, metabolic syndrome, and diabetes in the elderly. Am. J. Clin. Nutr..

[66] Jang Y., Lee J.H., Kim O.Y., Koh S.J., Chae J.S., Woo J.H., Cho H., Lee J.E., Ordovas J.M. (2006). The SNP276 G > T polymorphism in the adiponectin (ACDC) gene is more strongly associated with insulin resistance and cardiovascular disease risk than SNP45 T > G in nonobese/nondiabetic Korean men independent of abdominal adiposity and circulating plasma adiponectin. Metabolism.

[67] Alsaleh A., Crepostnaia D., Maniou Z., Lewis F.J., Hall W.L., Sanders T.A., O’Dell S.D., MARINA Study Team (2013). Adiponectin gene variant interacts with fish oil supplementation to influence serum adiponectin in older individuals. J. Nutr..

[68] Marín C., Perez-Jimenez F., Gomez P., Delgado J., Paniagua J.A., Lozano A., Cortes B., Jimenez-Gomez Y., Gomez M.J., Lopez-Miranda J. (2005). The Ala54Thr polymorphism of the fatty acid-binding protein 2 gene is associated with a change in insulin sensitivity after a change in the type of dietary fat. Am. J. Clin. Nutr..

[69] Fisher E., Schreiber S., Joost H.G., Boeing H., Döring F. (2011). A two-step association study identifies CAV2 rs2270188 single nucleotide polymorphism interaction with fat intake in type 2 diabetes risk. J. Nutr..

[70] Corella D., Qi L., Tai E.S., Deurenberg-Yap M., Tan C.E., Chew S.K., Ordovas J.M. (2006). Perilipin gene variation determines higher susceptibility to insulin resistance in Asian women when consuming a high-saturated fat, low-carbohydrate diet. Diabetes Care.

[71] Delgado-Lista J., Perez-Martinez P., García-Ríos A., Philipis C.M., Hall W., Gjelstad I.M.F., Lairon D., Saris W., Kiec-Wilk B., Karlström B. (2013). A gene variation (rs12691) in the CCAT/enhancer binding protein α modulates glucose metabolism in metabolic syndrome. Nutr. Metab. Cardiovasc..

[72] García-Ríos A., Gomez-Delgado F.J., Garaulet M., Alcala-Diaz J.F., Delgado-Lista F.J., Marin C., Rangel-Zúñiga O.A., Rodriguez-Cantalejo F., Gómez-Luna P., Ordovas J.M. (2014). Beneficial effect of CLOCK gene polymorphism rs1801260 in combination with low-fat diet on insulin metabolism in patients with metabolic syndrome. Chronobiol. Int..

[73] Dashti H.S., Smith C.E., Lee Y.C., Parnell L.D., Lai C.Q., Arnett D.K., Ordovas J.M., Garaulet M. (2014). CRY1 circadian gene variants interacts with carbohydrate intake for insulin resistance in two independent populations: Mediterranean and North American. Chronobiol. Int..

[74] Botden I.P., Zillikens M.C., de Rooij S.R., Langendonk J.G., Danser A.H., Sijbrands E.J., Roseboom T.J. (2012). Variants in the SIRT1 gene may affect diabetes risk in interaction with prenatal exposure to famine. Diabetes Care.

[75] Lazar M.A. (2005). PPAR gamma, 10 years later. Biochimie.

[76] Fisher E., Boeing H., Fritsche A., Doering F., Joost H.G., Schulze M.B. (2009). Whole-grain consumption and transcription factor-7-like 2 (TCF7L2) rs7903146: Gene-diet interaction in modulating type 2 diabetes risk. Br. J. Nutr..

[77] Florez J.C., Jablonski K.A., Bayley N., Pollin T.I., de Bakker P.I., Shuldiner A.R., Knowler W.C., Nathan D.M., Altshuler D., Diabetes Prevention Program Research Group (2006). TCF7L2 polymorphisms and progression to diabetes in the Diabetes Prevention Program. N. Engl. J. Med..

[78] Haupt A., Thamer C., Heni M., Ketterer C., Machann J., Schick F., Machicao F., Stefan N., Claussen C.D., Häring H.U. (2010). Gene variants of TCF7L2 influence weight loss and body composition during lifestyle intervention in a population at risk for type 2 diabetes. GDiabetes.

[79] Reinehr T., Friedel S., Mueller T.D., Toschke A.M., Hebebrand J., Hinney A. (2008). Evidence for an influence of TCF7L2 polymorphism rs7903146 on insulin resistance and sensitivity indices in overweight children and adolescents during a lifestyle intervention. Int. J. Obes..

[80] Chimienti F. (2013). Zinc, pancreatic islet function and diabetes: New insights into an old story. Nutr. Res. Rev..

[81] Shan Z., Bao W., Zhang Y., Rong Y., Wang X., Jin Y., Song Y., Yao P., Sun C., Hu F.B. (2014). Interactions between zinc transporter-8 gene (SLC30A8) and plasma zinc concentrations for impaired glucose regulation and type 2 diabetes. Diabetes.

[82] Marcheva B., Ramsey K.M., Buhr E.D., Kobayashi Y., Su H., Ko C.H., Ivanova G., Omura C., Mo S., Vitaterna M.H. (2010). Disruption of the clock components CLOCK and BMAL1 leads to hypoinsulinaemia and diabetes. Nature.

[83] Zheng J.S., Arnett D.K., Lee Y.C., Shen J., Parnell L.D., Smith C.E., Richardson K., Li D., Borecki I.B., Ordovás J.M. (2013). Genome-wide contribution of genotype by environment interaction to variation of diabetes-related traits. PLoS One.

[84] Babu P.V., Liu D., Gilbert E.R. (2013). Recent advances in understanding the anti-diabetic actions of dietary flavonoids. J. Nutr. Biochem..

[85] Wedick N.M., Pan A., Cassidy A., Rimm E.B., Sampson L., Rosner B., Willett W., Hu F.B., Sun Q., van Dam R.M. (2012). Dietary flavonoid intakes and risk of type 2 diabetes in US men and women. Am. J. Clin. Nutr..

[86] Hanhineva K., Torronen R., Bondia-Pons I., Pekkinen J., Kolehmainen M., Mykkanen H., Poutanen K. (2010). Impact of dietary polyphenols on carbohydrate metabolism. Int. J. Mol. Sci..

[87] Cai E.P., Lin J.K. (2009). Epigallocatechingallate (EGCG) and rutin suppress the glucotoxicity through activating IRS2 and AMPK signaling in rat pancreatic beta cells. J. Agric. Food Chem..

[88] Zhang Z., Ding Y., Dai X., Wang J., Li Y. (2011). Epigallocatechin-3-gallate protects pro-inflammatory cytokine induced injuries in insulin-producing cells through the mitochondrial pathway. Eur. J. Pharmacol..

[89] Ortsater H., Grankvist N., Wolfram S., Kuehn N., Sjoholm A. (2012). Diet supplementation with green tea extract epigallocatechingallate prevents progression to glucose intolerance in db/db mice. Nutr. Metab..

[90] Jung U.J., Lee M.K., Park Y.B., Kang M.A., Choi M.S. (2006). Effect of citrus flavonoids on lipid metabolism and glucose-regulating enzyme mRNA levels in type-2 diabetic mice. Int. J. Biochem. Cell Biol..

[91] Sharma A.K., Bharti S., Ojha S., Bhatia J., Kumar N., Ray R., Kumari S., Arya D.S. (2011). Up-regulation of PPARgamma, heat shock protein-27 and -72 by naringin attenuates insulin resistance, beta-cell dysfunction, hepatic steatosis and kidney damage in a rat model of type 2 diabetes. Br. J. Nutr..

[92] Takikawa M., Inoue S., Horio F., Tsuda T. (2010). Dietary anthocyanin-rich bilberry extract ameliorates hyperglycemia and insulin sensitivity via activation of AMP-activated protein kinase in diabetic mice. J. Nutr..

[93] Tsuda T., Horio F., Uchida K., Aoki H., Osawa T. (2003). Dietary cyanidin 3-*O*-beta-d-glucoside-rich purple corn color prevents obesity and ameliorates hyperglycemia in mice. J. Nutr..

[94] Kobori M., Masumoto S., Akimoto Y., Takahashi Y. (2009). Dietary quercetin alleviates diabetic symptoms and reduces streptozotocin-induced disturbance of hepatic gene expression in mice. Mol. Nutr. Food Res..

[95] Kim E.K., Kwon K.B., Song M.Y., Han M.J., Lee J.H., Lee Y.R., Ryu D.G., Park B.H., Park J.W. (2007). Flavonoids protect against cytokine-induced pancreatic beta-cell damage through suppression of nuclear factor kappa B activation. Pancreas.

[96] Fu Z., Zhang W., Zhen W., Lum H., Nadler J., Bassaganya-Riera J., Jia Z., Wang Y., Misra H., Liu D. (2010). Genistein induces pancreatic beta-cell proliferation through activation of multiple signaling pathways and prevents insulin-deficient diabetes in mice. Endocrinology.

[97] Castellano J.M., Guinda A., Delgado T., Rada M., Cayuela J.A. (2013). Biochemical basis of the antidiabetic activity of oleanolic acid and related pentacyclic triterpenes. Diabetes.

[98] Zhao H.L., Sui Y., Qiao C.F., Yip K.Y., Leung R.K., Tsui S.K., Lee H.M., Wong H.K., Zhu X., Siu J.J. (2012). Sustained antidiabetic effects of a berberine-containing Chinese herbal medicine through regulation of hepatic gene expression. Diabetes.

[99] Chatuphonprasert W., Lao-Ong T., Jarukamjorn K. (2013). Improvement of superoxide dismutase and catalase in streprozotocin-nicotinamide-induced type 2 diabetes in mice by berberine and glibenclamide. Pharm. Biol..

[100] Gysemans C.A., Cardozo A.K., Callewaert H., Giulietti A., Hulshagen L., Bouillon R., Eizirik D.L., Mathieu C. (2005). 1,25-Dihydroxyvitamin D_3_ modulates expression of chemokines and cytokines in pancreatic islets: Implications for prevention of diabetes in nonobese diabetic mice. Endocrinology.

[101] Wolden-Kirk H., Rondas D., Bugliani M., Korf H., van lommel L., Brusgaard K., Christesen H.T., Schuit F., Proost P., Masini M. (2014). Discovery of molecular pathways mediating 1,25-dihydroxyvitamin D_3_ protection against cytokine-induced inflammation and damage of human and male mouse islets of Langerhans. Endocrinology.

[102] Lazo de la Vega-Monroy M.L., Larrieta E., German M.S., Baez-Saldana A., Fernandez-Mejia C. (2013). Effects of biotin supplementation in the diet on insulin secretion, islet gene expression, glucose homeostasis and beta-cell proportion. J. Nutr. Biochem..

[103] Cobianchi L., Fornoni A., Pileggi A., Molano R.D., Sanabria N.Y., Gonzalez-Quintana J., Bocca N., Marzorati S., Zahr E., Hogan A.R. (2008). Riboflavin inhibits IL-6 expression and p38 activation in islet cells. Cell Transplant..

[104] Ye D.Z., Tai M.H., Linning K.D., Szabo C., Olson L.K. (2006). MafA expression and insulin promoter activity are induced by nicotinamide and related compounds in INS-1 pancreatic beta-cells. Diabetes.

[105] Xu G., Kwon G., Cruz W.S., Marshall C.A., McDaniel M.L. (2001). Metabolic regulation by leucine of translation initiation through the mTOR signaling pathway by pancreatic beta-cells. Diabetes.

[106] Carneiro E.M., Latorraca M.Q., Araujo E., Beltrá M., Oliveras M.J., Navarro M., Berná G., Bedoya F.J., Velloso L.A., Soria B. (2009). Taurine supplementation modulates glucose homeostasis and islet function. J. Nutr. Biochem..

[107] Corless M., Kiely A., McClenaghan N.H., Flatt P.R., Newsholme P. (2006). Glutamine regulates expression of key transcription factor, signal transduction, metabolic gene, and protein expression in a clonal pancreatic beta-cell line. J. Endocrinol..

[108] Ritz-Laser B., Meda P., Constant I., Klages N., Charollais A., Morales A., Magnan C., Ktorza A., Philippe J. (1999). Glucose-induced preproinsulin gene expression is inhibited by the free fatty acid palmitate. Endocrinology.

[109] Hagman D.K., Hays L.B., Parazzoli S.D., Poitout V. (2005). Palmitate inhibits insulin gene expression by altering PDX-1 nuclear localization and reducing MafA expression in isolated rat islets of Langerhans. J. Biol. Chem..

[110] Qiu L., List E.O., Kopchick J.J. (2005). Differentially expressed proteins in the pancreas of diet-induced diabetic mice. Mol. Cell Proteomics.

[111] Dreja T., Jovanovic Z., Rasche A., Kluge R., Herwig R., Tung Y.C., Joost H.G., Yeo G.S., Al-Hasani H. (2010). Diet-induced gene expression of isolated pancreatic islets from a polygenic mouse model of the metabolic syndrome. Diabetologia.

[112] Kluth O., Mirhashemi F., Scherneck S., Kaiser D., Kluge R., Neschen S., Joost H.G., Schürmann A. (2011). Dissociation of lipotoxicity and glucotoxicity in a mouse model of obesity associated diabetes: Role of forkhead box O1 (FOXO1) in glucose-induced beta cell failure. Diabetologia.

[113] Castro M.C., Francini F., Gagliardino J.J., Massa M.L. (2014). Lipoic acid prevents fructose-induced changes in liver carbohydrate metabolism: Role of oxidative stress. Biochim. Biophys. Acta.

[114] Obici S., Feng Z., Morgan K., Stein D., Karkanias G., Rossetti L. (2002). Central administration of oleic acid inhibits glucose production and food intake. Diabetes.

[115] Morgan K., Obici S., Rossetti L. (2004). Hypothalamic responses to long-chain fatty acids are nutritionally regulated. J. Biol. Chem..

[116] Jäger S., Trojan H., Kopp T., Laszczky M.N., Scheffler A. (2009). Pentacyclictriterpene distribution in various plant-rich sources for a new group of multi-potent plant extracts. Molecules.

[117] Guinda A., Rada M., Delgado T., Gutierrez-Adane P., Castellano J.M. (2010). Pentacyclictriterpenoids from olive fruit and leaf. J. Agric. Food Chem..

[118] Dong H., Wang N., Zhao L., Lu F. (2012). Berberine in the treatment of type 2 diabetes mellitus: A systemic review and meta-analysis. Evid. Based Complement. Altern. Med..

[119] De Oliveira B.F., Costa D.C., Nogueira-Machado J.A., Chaves M.M. (2013). β-Carotene, α-tocopherol and ascorbic acid: Differential profile of antioxidant, inflammatory status and regulation of gene expression in human mononuclear cells of diabetic donors. Diabetes Metab. Rev. Res..

[120] De Oliveira C.A., Latorraca M.Q., de Mello M.A., Carneiro E.M. (2011). Mechanisms of insulin secretion in malnutrition: Modulation by amino acids in rodent models. Amino Acids.

[121] Dickson L.M., Rhodes C.J. (2004). Pancreatic beta-cell growth and survival in the onset of type 2 diabetes: A role for protein kinase B in the Akt?. Am. J. Physiol. Endocrinol. Metab..

[122] Kwon G., Marshall C.A., Pappan K.L., Remedi M.S., McDaniel M.L. (2004). Signaling elements involved in the metabolic regulation of mTOR by nutrients, incretins, and growth factors in islets. Diabetes.

[123] Newsholme P., Brennan L., Rubi B., Maechler P. (2005). New insights into amino acid metabolism, beta-cell function and diabetes. Clin. Sci..

[124] Newsholme P., Bender K., Kiely A., Brennan L. (2007). Amino acid metabolism, insulin secretion and diabetes. Biochem. Soc. Trans..

[125] Lindström J., Ilanne-Parikka P., Peltonen M., Aunola S., Eriksson J.G., Hemiö K., Hämäläinen H., Härkönen P., Keinänen-Kiukaanniemi S., Laakso M. (2006). Sustained reduction in the incidence of type 2 diabetes by lifestyle intervention: Follow-up of the Finnish Diabetes Prevention Study. Lancet.

[126] Imai Y., Patel H.R., Doliba N.M., Matschinsky F.M., Tobias J.W., Ahima R.S. (2008). Analysis of gene expression in pancreatic islets from diet-induced obese mice. Physiol. Genomics.

[127] Marchetti P., Bugliani M., Boggi U., Masini M., Marselli L. (2012). The pancreatic beta cells in human type 2 diabetes. Adv. Exp. Med. Biol..

[128] Udupa A., Nahar P., Shah S., Kshirsagar M., Ghongane B. (2013). A comparative study of effects of omega-3 fatty acids, alpha lipoic acid and vitamin E in type 2 diabetes mellitus. Ann. Med. Health Sci. Res..

[129] Igoillo-Esteve M., Marselli L., Cunha D.A., Ladrière L., Ortis F., Grieco F.A., Dotta F., Weir G.C., Marchetti P., Eizirik D.L. (2010). Palmitate induces a pro-inflammatory response in human pancreatic islets that mimics CCL2 expression by beta cells in type 2 diabetes. Diabetologia.

[130] Choi H.J., Hwang S., Lee S.H., Lee Y.R., Shin J., Park K.S., Cho Y.M. (2012). Genome-wide identification of palmitate-regulated immediate early genes and target genes in pancreatic beta-cells reveals a central role of NF-κB. Mol. Biol. Rep..

[131] Kharroubi I., Ladrière L., Cardozo A.K., Dogusan Z., Cnop M., Eizirik D.L. (2004). Free fatty acids and cytokines induce pancreatic beta-cell apoptosis by different mechanisms: Role of nuclear factor-kappaB and endoplasmic reticulum stress. Endocrinology.

[132] Konstantinidou V., Khymenets O., Covas M.I., de la Torre R., Muñoz-Aguayo D., Anglada R., Farré M., Fito M. (2009). Time course of changes in the expression of insulin sensitivity-related genes after an acute load of virgin olive oil. OMICS.

[133] Kallio P., Kolehmainen M., Laaksonen D.E., Kekäläinen J., Salopuro T., Sivenius K., Pulkkinen L., Mykkänen H.M., Niskanen L., Uusitupa M. (2007). Dietary carbohydrate modification induces alterations in gene expression in abdominal subcutaneous adipose tissue in persons with the metabolic syndrome: The FUNGENUT Study. Am. J. Clin. Nutr..

[134] Crujeiras A.B., Parra D., Milagro F.I., Goyenechea E., Larrarte E., Margareto J., Martínez J.A. (2008). Differential expression of oxidative stress and inflammation related genes in peripheral blood mononuclear cells in response to a low-calorie diet: A nutrigenomics study. OMICS.

[135] Okamoto H., Nakae J., Kitamura T., Park B.C., Dragatsis I., Accili D. (2004). Transgenic rescue of insulin receptor-deficient mice. J. Clin. Investig..

[136] Bruning J.C., Gautam D., Burks D.J., Gillette J., Schubert M., Orban P.C., Klein R., Krone W., Müller-Wieland D., Kahn C.R. (2000). Role of brain insulin receptor in control of body weight and reproduction. Science.

[137] Duca F.A., Yue J.T. (2014). Fatty acid sensing in the gut and the hypothalamus: *In vivo* and *in vitro* perspectives. Mol. Cell Endocrinol..

[138] Fick L.J., Belsham D.D. (2010). Nutrient sensing and insulin signaling in neuropeptide-expressing immortalized, hypothalamic neurons. Cell Cycle.

[139] Bird A. (2007). Perceptions of epigenetics. Nature.

[140] Ambors V. (2004). The function of animals micro RNAs. Nature.

[141] Kim D.H., Saetrom P., Snove O., Rossi J.J. (2008). MicroRNA-directed transcriptional gene silence in mammalian cells. Proc. Natl. Acad. Sci. USA.

[142] García-Segura L., Peréz-Andrade M., Miranda-Ríos J. (2013). The emerging role of micro RNAs in the regulation of gene expression by nutrients. J. Nutrigenet. Nutrigenomics.

[143] Roseboom T., de Rooij S., Painter R. (2006). The Dutch famine and its long-term consequences for adult health. Early Hum. Dev..

[144] De Rooij S.R., Painter R.C., Roseboom T.J., Phillips D.I., Osmond C., Barker D.J., Tanck M.W., Michels R.P., Bossuyt P.M., Bleker O.P. (2006). Glucose tolerance at age 58 and the decline of glucose tolerance in comparison with age 50 in people prenatally exposed to the Dutch famine. Diabetologia.

[145] Ng S.F., Lin R.C., Laybutt D.R., Barres R., Owens J.A., Morris M.J. (2010). Chronic high-fat diet in fathers programs beta-cell dysfunction in female rat offspring. Nature.

[146] Inoue T., Kido Y., Ashara S., Matsuda T., Shibutani Y., Koyanagi M., Kasuga M. (2009). Effect of intrauterine undernutrition during late gestation on pancreatic beta cell mass. BioMed. Res..

[147] Volkmar M., Dedeurwaerder S., Cunha D.A., Ndlovu M.N., Defrance M., Deplus R., Calonne E., Volkmar U., Igoillo-Esteve M., Naamane N. (2012). DNA methylation profiling identifies epigenetic dysregulation in pancreatic islets from type 2 diabetic patients. EMBO J..

[148] Fernández-Valverde S.L., Taft R.J., Mattick J.S. (2011). Micro RNAs in β cell biology, insulin resistance, diabetes and its complications. Diabetes.

[149] Frost R.J.A., Olson E.N. (2011). Control of glucose homeostasis and insulin sensitivity by the Let-7 family of micro RNAs. Proc. Natl. Acad. Sci. USA.

[150] Bladé C., Baselga-Escudero L., Salvadó M.J., Arola-Arnal A. (2013). miRNAS, polyphenols and chronic disease. Mol. Nutr. Food Res..

[151] Palmer J.D., Soule B.P., Simone B.A., Zaorsky N.G., Jin L., Simone N.L. (2014). Micro RNA expression altered by diet: Can food be medicinal?. Ageing Res. Rev..

